# Evaluation of Salivary Function Post-Partial Superficial Parotidectomy

**DOI:** 10.22038/IJORL.2024.76003.3551

**Published:** 2024-05

**Authors:** Sona Sepahi, Atena Aghaee, Imaneh Roshanzamir, Adel Ghorani-Azam, Soheila Erfani, Leila Mashhadi, Kamran Khazaeni

**Affiliations:** 1 *Department of Otorhinolaryngology, Mashhad University of Medical Sciences, Mashhad, Iran. *; 2 *Nuclear Medicine Research Center, Mashhad University of Medical Sciences, Mashhad, Iran.*; 3 * Sinus and Surgical Endoscopic Research Center, Mashhad University of Medical Sciences, Mashhad, Iran.*; 4 *Department of Forensic Medicine and Toxicology, School of Medicine, Urmia University of Medical Sciences, Urmia, Iran.*; 5 *Department of Anesthesiology and Intensive Care, Mashhad University of Medical Sciences, Mashhad, Iran.*

**Keywords:** Parotid Pleomorphic Adenomas, Partial Superficial Parotidectomy, Salivary Function Preservation, Technetium-99m Scintigraphy

## Abstract

**Introduction:**

Parotid pleomorphic adenomas necessitate surgical intervention, with a growing emphasis on preserving salivary function post-surgery due to its critical role in maintaining oral health and overall quality of life. This study aims to evaluate a surgical method meticulously designed to preserve salivary function following partial superficial parotidectomy, utilizing Technetium-99m scintigraphy.

**Materials and Methods:**

This single-center prospective cohort study was conducted in Mashhad, Iran, between 2022 and 2023. The study encompassed 40 patients diagnosed with parotid pleomorphic adenomas, ages 20 to 64, undergoing partial superficial parotidectomy. The salivary function was evaluated using Technetium-99m scintigraphy three weeks post-operation.

**Results:**

Most participants underwent right parotid surgery (62.5%, n=25) instead of left parotid surgery (37.5%, n=15). The outcomes of the partial superficial parotidectomy indicated no complications during the three-week post-operative period. Saliva secretion rates on the operated side were preserved across the cohort. A significant difference in saliva secretion rates was observed between the operated and contralateral sides (P<0.01) for both right and left parotid surgery groups. No significant correlation was found between the time elapsed post-surgery and saliva secretion rates (P=0.48).

**Conclusion:**

Our study demonstrated that the superficial parotidectomy technique is notably effective when focused on preserving the salivary function of the deep parotid gland. Not only does it maintain saliva secretion on the operated side, but it also boasts an admirable safety profile. There were no recorded complications, and duct preservation was achieved in most instances.

## Introduction

Parotid pleomorphic adenomas are benign neoplasms predominantly originating from the epithelial elements of the parotid gland, the largest of the salivary glands (1,2). Despite their benign nature, they can exhibit expansive growth, leading to facial deformity and potential malignant transformation over time, thereby necessitating surgical intervention (3). Surgical removal remains the cornerstone of management for parotid pleomorphic adenomas. 

The predominant surgical modality employed for such cases is the superficial parotidectomy. This technique necessitates the excision of the entirety of the superficial lobe of the parotid gland. One notable complication is the ligation of the Stensen's duct, which can precipitate salivary dysfunction. Notably, even if secretory activity within the deep lobe remains unaltered, the absence of an adequate drainage conduit can impede salivary flow (4-6).

In recent years, there has been a growing emphasis on preserving the salivary function post-surgery, given its critical role in maintaining oral health and overall quality of life (7). Salivary glands play a pivotal role in oral homeostasis, aiding digestion, protecting against microbial infections, and facilitating oral tissue repair (8). Therefore, preserving these functions is vital in the surgical management of parotid pleomorphic adenomas.

In this context, our study seeks to assess a refined surgical technique for superficial parotidectomy, meticulously crafted to preserve salivary function following a partial excision of the superficial parotid without involving or exposing Stensen's duct. Our objective is to present a thorough evaluation of post-operative outcomes using Technetium-99m scintigraphy as a diagnostic measure for salivary gland function (9). This investigation aims to provide significant insights to further the ongoing endeavors to optimize the efficacy and safety of surgical treatments for parotid pleomorphic adenomas.

## Materials and Methods

Study Design and Patient Selection

This prospective cohort study was conducted between 2022 and 2023in the Otolaryngology-Head and Neck Surgery Department, Qaem Hospital, Mashhad University of Medical Sciences, Mashhad, Iran. The study included patients with confirmed parotid pleomorphic adenomas who were candidates for partial superficial parotidectomy.

*Inclusion and Exclusion Criteria*

The inclusion criteria were individuals aged 18-65, confirmed parotid pleomorphic adenoma diagnosis in histopathologic evaluation, suitability for parotidectomy as per guidelines, and absence of previous parotid surgery. The major exclusion criteria were malignant parotid carcinomas, parotid duct ligation during surgery, and the absence of secretory activity of the gland in the post-operative scintigraphy. Additionally, individuals who withdraw from the study plan, fail to complete informed consent, or with underlying diseases influencing the level of salivary secretion, consuming drugs that affect salivary secretion (such as anticholinergics and Tricyclic antidepressants), and individuals with a history of previous parotidectomy.

*Data Collection*

The gathered data included demographic details, age, past medical history, the type of parotid surgery (whether right or left), Technetium-99 uptake levels, the interval between surgery and scanning, salivary secretion rates following parotidectomy, and follow-up outcomes, specifically complications. Additionally, the status of duct preservation was documented, whether it was successfully identified and preserved during the surgery and/or showed secretions in the post-operative scintigraphy.

*Partial* *superficial* *parotidectomy* *surgical technique *

First, the patient was positioned flat on their back on the operating table, with their head turned to the side opposite to where the surgery was to be conducted. This positioning facilitated a clear view and easy neck and parotid region access. Subsequently, a gentle incision was made at the front of the ear, extending it downwards to follow the skin’s natural lines, a technique known to reduce visible scarring post-surgery. This incision, often referred to as a modified Blair or facelift incision, served as the entry point to the affected area. Following the incision, the skin flap was carefully elevated to reveal the outer portion of the parotid gland, setting the stage for the critical steps of the surgery ahead. The identification and preservation of the facial nerve, which traverses the parotid gland, emerged as a crucial part of the procedure. The nerve was successfully protected with specialized nerve monitoring equipment and a detailed dissection process, preventing potential damage. After this, attention was turned to the gland dissection, separating it from the surrounding structures and identifying the part of the gland that harbored the lesion. This step was essential in preparing the affected area for removal. The identified portion of the gland was then removed, with a concerted effort to preserve as much of the healthy glandular tissue as possible, thereby ensuring en bloc tumor removal with a cuff of parotid tissue to avoid invasion of the tumor capsule and the risk of recurrence. An attempt was made to find and preserve the Stensen’s duct at the anterior border of the gland at the entrance to the masseter muscle. In cases where the duct was not found, the preservation of the duct was proven by the secretory activity of the gland in the post-operative scintigraphy. Precise techniques involving electrocautery or ligatures were employed to secure the area and maintain a clean surgical site to avert any post-operative bleeding. As the procedure concluded, the wound was closed in layers, with the skin being aligned carefully to minimize any scarring and foster smooth healing. Lastly, to prevent any fluid accumulation in the surgical area, a drain was placed, aiding in removing any excess fluids and averting complications such as swelling or hematoma formation.

*Technetium-99m(Tc-99m) Scintigraphy parotid salivary gland Protocol*

By the procedure 18 to 200 days after surgery, the patients who had fasted for 4 hours were positioned with their heads in the Waters projection (a frontal viewpoint with the head reclined at a 45-degree angle). 

Following this, Tc-99m pertechnetate was administered through an intravenous injection, and sequential images were acquired (comprising 240 frames at 15-second intervals during the uptake phase). When the 180th frame is reached (marking the conclusion of the uptake phase), lemon juice is ingested to facilitate saliva production (initiating the discharge phase). Established quantitative benchmarks for both uptake and discharge phases are available for the parotid and submandibular glands.

*Statistical analysis and sample size calculation*

The gathered data were examined using the SPSS statistical software (version 26).

Descriptive statistics were used to describe the demographic characteristics of the study population, including mean and standard deviation calculations for age and percentages for gender distribution. Wilcoxon rank sum tests were employed to compare the saliva secretion rates between the operated and contralateral sides in both the right and left parotid surgery groups. Also, Wilcoxon rank sum tests were used to analyze the relationship between the time elapsed post-surgery and saliva secretion rates. For all statistical tests, p-values less than 0.05 were considered statistically significant. Participants were grouped according to the affected side of the parotid gland, as this approach allows for a more precise analysis of the surgical impact on salivary gland function, considering the possibility of inherent biases towards one side due to anatomical differences or surgeon’s preference, thereby enhancing the validity and applicability of the study findings. The determination of the sample size for this investigation was predicated on an analysis of previous similar studies on this patient cohort, specifically focusing on the percentual decrement observed in the Confidence Interval (CI) ratio (10). The threshold for the minimum clinically significant effect was established at 58%. The requisite sample size was computed using Sigma Plot, accounting for a standard deviation 1.2 and an intervention effect culminating in an 84% CI percentage ratio. Adhering to a significance level (α) of 0.05 and targeting a statistical power of 90%, the calculation yielded a total sample size of 40 subjects. 

## Results

*Demographic Characteristics of the Study Population*

In the present study, 40 patients were enrolled, comprising 27 women (67.5%) and 13 men (32.5%). The participants exhibited a mean age of 42.92 ± 11.02 years, spanning an age range of 20 to 64 years.

*Clinical Observations and Surgical Details*

The cohort predominantly consisted of individuals who underwent right parotid surgery (62.5%, n=25) as opposed to left parotid surgery (37.5%, n=15). A comprehensive review of the patients' medical histories revealed that 33 individuals had pre-existing conditions, including hypothyroidism (5.0%, n=2) and hypertension (12.5%, n=5). Notably, 32 participants (80.0%) had no prior surgical history, while 8 (20.0%) reported previous cataract and orthopedic surgeries. Importantly, no participants exhibited conditions potentially impacting salivary function, such as Sjogren's syndrome.

Medication History and Technetium-99 Uptake

All participants demonstrated Technetium-99 uptake during the study. Medication histories were largely unremarkable, with no individuals on medications known to affect saliva secretion. 

*Saliva Secretion Rates and Surgical Timing*

**Fig 1:**
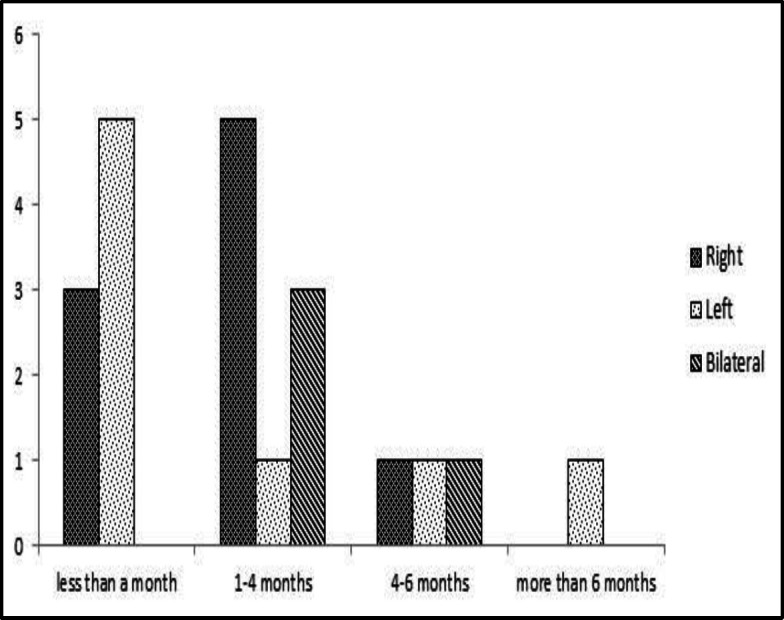
Comparison of the time interval between the surgery and the scan in patients who had abnormal saliva secretion

The average interval between surgery and scanning was 95.04 ± 61.34 days, ranging from 18 to 200 days. Statistical analysis and Wilcoxon rank sum tests revealed no significant correlation between the time elapsed post-surgery and saliva secretion rates (P=0.48) (Fig. 1). 

*Saliva Secretion Analysis Post-Parotidectomy*

In the right parotid surgery group, nine patients exhibited decreased saliva secretion (<28%), while 16 demonstrated normal levels (>28%). The mean saliva secretion rates were 26.24 ± 11.99% on the operated side and 42.28 ± 11.54% on the contralateral side, a statistically significant difference (p<0.01). Conversely, in the left parotid surgery group, abnormal and normal saliva secretion was observed in 8 and 7 patients, respectively. The mean secretion rates were 19.06 ± 11.01% on the operated side and 39.26 ± 14.17% on the contralateral side, a significant difference (p<0.01). Notably, four patients exhibited abnormal saliva secretion in both parotids post-surgery, with three having undergone right parotidectomy and one left parotidectomy. In addition, in comparison means of operating side together, there was no significant difference across sides (p = 0.09). 

*Post-Operative* *Follow-Up* *and* *Duct Preservation*

Post-operative follow-ups from 18 to 200 days indicated no complications such as sialocele or recurrence, affirming the efficacy of partial superficial parotidectomy in achieving complete lesion resolution. Furthermore, the parotid gland duct was successfully identified and preserved in 23 cases (57.5%), with no evidence of duct damage in the remaining 17 cases (42.5%) where the duct was not identified during the surgery. However, preservation was confirmed by salivary secretion in post-operative scintigraphy.

## Discussion

In the present study, a cohort of 40 patients was closely examined, with a significant portion undergoing right parotid surgery (62.5%). Most participants had no substantial medical history that might influence the saliva secretion rates. A case of particular interest involved a 57-year-old female who experienced a marked reduction in saliva secretion following the surgical procedure. The data revealed a notable decline in saliva secretion rates on the operated side, compared to the contralateral side, across both right and left parotid surgery groups. 

A minority of patients demonstrated irregular saliva secretion in both parotids post-operatively. Subsequent follow-ups post-surgery demonstrated favorable outcomes, with no complications, suggesting the preservation of the parotid gland duct in most cases and thereby affirming the effectiveness of the surgical procedure in retaining gland functionality. In the broader context of recent research, studies have endeavored to delineate the most efficacious and safe surgical interventions for managing parotid pleomorphic adenomas. In comparing our manuscript with the Park study, we note that Park's research highlights the efficacy of extracapsular dissection (ECD) over partial superficial parotidectomy (PSP) and classic superficial parotidectomy (CSP) for preserving salivary function after benign parotid tumor surgeries. It emphasizes ECD's advantages in reduced operative times, complications, and better post-operative salivary flow rates (11).

Conversely, our study, focusing on a meticulous PSP technique verified through Technetium-99m scintigraphy, underscores the effectiveness and safety of PSP in preserving salivary secretion without significant complications. The difference in outcomes can be attributed to our study's concentrated effort on refining PSP techniques specifically for salivary preservation instead of Park's broader comparison across different surgical methods. Our specific focus on PSP offers a complementary perspective to Park's work, suggesting that with refined techniques, PSP can achieve favorable outcomes in salivary function preservation following parotidectomy for benign tumors. The study by Zhang et al. (2013) (12) explored the outcomes of partial superficial parotidectomy (PP) versus total superficial parotidectomy (TP) in terms of facial nerve function and salivary secretion. Zhang et al. demonstrated that PP significantly reduces the incidence of post-operative facial nerve dysfunction and is conducive to preserving Stensen's duct and saliva secretion, aligning with our findings that meticulous surgical techniques in PSP can effectively maintain salivary function. However, our study further refines the approach by using Technetium-99m scintigraphy to objectively measure post-operative salivary gland function, offering a nuanced perspective on the efficacy of PSP. 

This difference in methodology and the inclusion of a more recent patient cohort in our study provide a contemporary validation of PSP's benefits, emphasizing its role in minimizing surgical morbidity while ensuring glandular preservation. The alignment of outcomes between our study and Zhang et al.'s work underlines the consistency of PSP's advantages over more invasive procedures, reinforcing the importance of technique refinement and surgical expertise in optimizing patient outcomes. Li's research, encompassing 156 patients undergoing partial superficial parotidectomy, underscored the safety of this technique, characterized by an absence of permanent facial nerve paralysis and a minimal recurrence rate. While the study accentuated the aesthetic satisfaction attained through this approach, a more comprehensive comparative analysis with alternative surgical techniques could strengthen the conclusions drawn (13). 

Conversely, Cristofaro's study adopted a comparative methodology, evaluating the outcomes of 198 patients who underwent either extracapsular dissection (ED) or superficial parotidectomy (SP). The research, which lasted nearly a decade, favored ED as a more desirable method, attributing its fewer adverse effects and comparable efficacy to SP (14).

Qin's research embarked on a retrospective analysis involving 75 patients, juxtaposing the impacts of partial superficial parotidectomy (PSP) and SP. This investigation highlighted the benefits of PSP, including reduced operative durations and heightened post-operative satisfaction, albeit limited by a smaller sample size and the necessity for extended follow-up periods (15). Furthermore, Silvoniemi's study undertook a longitudinal analysis, scrutinizing data from patients treated between 1979 and 1996. Although revealing a relatively low recurrence rate, it indicated a significant proportion of patients experiencing post-operative facial nerve dysfunction (16). Lastly, Ogreden's research focused on comparing the incidence rates of Frey syndrome following superficial parotidectomy and partial superficial parotidectomy. No significant disparity was discerned in a cohort of 50 patients between the two techniques concerning the development of Frey syndrome and recurrence rates. Nevertheless, expanding the sample size and prolonged follow-up might furnish more definitive insights (17). 

In scrutinizing the design and execution of our prospective cohort study, several noteworthy limitations emerge. Firstly, the single-center nature of the study potentially narrows the scope of the findings, making it less reflective of diverse patient populations and surgical approaches that might be encountered in a multi-center study. This aspect might limit the generalizability of the results, confining the insights to a specific demographic and geographic locale. 

Furthermore, the study encompasses a relatively short follow-up period. This truncated timeframe might need to be sufficiently expansive to fully capture the long-term outcomes and recurrence rates, potentially overlooking delayed complications or gradual improvements in salivary function. Moreover, the study grapples with the potential for observing small effect sizes, which might not hold substantial clinical significance. This scenario could lead to an overestimation of the impact of the surgery on salivary function, thereby not offering a comprehensive view of the potential benefits and drawbacks of the surgical intervention.

## Conclusions

In conclusion, our study demonstrated that the superficial parotidectomy technique is notably effective when focused on preserving the salivary function of the deep parotid gland. Not only does it maintain saliva secretion on the operated side, but it also boasts an admirable safety profile. There were no recorded complications, and duct preservation was achieved in most of the instances.
